# Coupling and interaction mechanism between green urbanization and tourism competitiveness based an empirical study in the Yellow River Basin of China

**DOI:** 10.1038/s41598-024-64164-8

**Published:** 2024-06-07

**Authors:** Wei Shen, Yanli Chen, Weiwei Cao, Ruyi Yu, Jinlong Cheng

**Affiliations:** 1 College of Geography and Tourism, Luoyang Normal University, Luoyang, 471022 China; 2 College of Law and Sociology, Luoyang Normal University, Luoyang, 471022 China; 3 College of Geography and Environmental Science, Henan University, Kaifeng, 475004 China; 4 Key Laboratory of Geospatial Technology for the Middle and Lower Yellow River Regions, Henan University, Kaifeng, 475004 China

**Keywords:** Green urbanization, Tourism competitiveness, Spatial coupling relation, Interactive mechanism, Coordinated development strategies, Environmental sciences, Environmental social sciences

## Abstract

Exploring the spatial coupling relationship and interaction mechanism between green urbanization (GU) and tourism competitiveness (TC) is of great significance for promoting urban sustainable development. However, the lack of research on the interaction mechanism between GU and TC limits the formulation of effective environmental management policy and urban planning. Taking 734 counties in the Yellow River Basin (YRB) as the study area, this paper analyzes the spatial coupling relationship between GU and TC on the basis of comprehensive evaluation of GU and TC. Then, the interactive mechanism between GU and TC is systematically discussed, and the synergistic development strategy of the two is proposed. The results show that the GU level presents a multicore circle structure, with provincial capitals, prefecture-level urban districts and economically developed counties in east-central regions as high-value centers. The TC at county scale presents a multi-center spatial structure. Additionally, there is a significant positive spatial coupling between GU and TC in the YRB. The analysis further reveals that green urbanization level, social progress, population development, infrastructure construction, economic development quality, and eco-environmental protection has a observably influence on TC. Tourism competitiveness, service competitiveness, location competitiveness, resource competitiveness, market competitiveness, environmental influence, and talent competitiveness has a observably influence on GU. TC can promote GU, and the improvement of green urbanization level can support the development of tourism competitiveness. According to the spatial zoning method, 734 counties are divided into 6 categories, and the coordinated development strategy of GU and TC for each type of district is proposed.

## Introduction

Since the reform and opening up, China's urbanization has developed rapidly. However, the extensive urbanization model characterized by high expansion, high consumption and high emissions has brought about a series of problems, such as environmental pollution, ecological destruction, disorderly expansion of urban space, unbalanced urban–rural and regional development, and unreasonable industrial structure^1,2^. Green urbanization emphasizes the transformation from rural to urban areas in terms of industrial support, living environment, social security and lifestyle, as well as the improvement of the quality of economic development, ecological environmental protection and social progress^3,4^. This makes city managers gradually realize that the implementation and promotion of green urbanization can help solve the negative problems caused by traditional urbanization. At the same time, with the advent of the era of mass leisure, tourism has become the pillar industry of county social and economic development and the key to urban green and low-carbon development and industrial transformation. Tourism competitiveness is the reflection of the overall development strength of regional tourism, and is also the core driving force for the development of tourism industry^5,6^. Therefore, green urbanization and tourism competitiveness are consistent in many aspects such as development concepts and goals. On the one hand, the improvement of tourism competitiveness not only provides a major opportunity for the sustainable development of regional tourism, but also releases a strong driving force for the promotion of green urbanization. On the other hand, green urbanization provides support for the improvement of tourism competitiveness. Green urbanization and tourism competitiveness complement each other and can jointly promote regional sustainable development. Therefore, in-depth research on the spatial coupling relationship and interaction mechanism between green urbanization and tourism competitiveness has important theoretical significance, and also has important practical significance for promoting urban sustainable development.

With the deepening of urbanization research, " green urbanization", which pays attention to the improvement of green development and urbanization quality, has gradually become the focus of academic research. At present, the research on green urbanization mainly focuses on five aspects: theoretical research^4,6^, evaluation method^7–10^, influencing factor^4,8–10^, dynamic mechanism^11^, and development path^12–15^. In terms of the research on the tourism competitiveness, the term "tourism competitiveness" began to appear in the 1990s^16–18^. Subsequently, after nearly 20 years of development, the theoretical research results of tourism competitiveness have been gradually enriched and a theoretical framework for the study of tourism competitiveness has been initially formed, which has laid a certain foundation for the subsequent exploration of quantitative research methods of tourism competitiveness. In terms of the assessment methods of tourism competitiveness, the index system evaluation method has been adopted in most of the existing studies^19–21^, and the evaluation indicators are mainly selected from five aspects: social and economic development^20–22^, tourism market development^6,20–22^, traffic and location conditions^21,22^, ecological environment^20–22^, and tourism resources^17,18,20^. In terms of research scale, existing research mainly carries out tourism competitiveness evaluation at provincial, city, and county scale based on social and economic statistical data^16,22^. In terms of the coupling relationship between green urbanization and tourism competitiveness, most studies have used bivariate spatial autocorrelation model and coupling coordination degree model to analyze the coupling relationship between green urbanization and tourism resource conversion efficiency^23^, the coupling relationship between green urbanization and tourism eco-efficiency^24^, the coupling coordination relationship between low-carbon cities and tourism development^25^, the coupling relationship between urbanization and tourism development^26^, the coupling relationship between tourism urbanization and eco-environmental quality^27,28^, and the coupling relationship between tourism development, urbanization and eco-environment^29^. For example, Hao et al. (2022) used SBM model, coupled coordination degree model, Tobit regression and other methods to explore the spatio-temporal evolution characteristics and influencing factors of coupled coordination degree (CCD) between new urbanization and tourism resource conversion efficiency in the Yellow River Basin^23^. Yang et al. (2022) evaluated the coordination relationship between green urbanization and sustainable tourism development from the perspective of decoupling coordination, and analyzed the spatio-temporal characteristics of the two^24^. Wang et al. (2019) used the Coupled coordination degree model (CCDM) to conduct an empirical study on the coupled coordination between low-carbon cities and tourism industry^25^. However, few studies have focused on the spatial coupling relationship between county green urbanization and tourism competitiveness. In terms of the interaction mechanism between green urbanization and tourism competitiveness, there have been more studies on the driving mechanism of green urbanization, and the driving mechanism of tourism competitiveness, but few studies have in-depth analysis of the interaction mechanism between green urbanization and tourism competitiveness. In terms of coordinated development strategy, a few scholars have proposed a coordinated development strategy between tourism competitiveness and green urbanization based on their personal research experience^30,31^, but less proposed differentiated regulation strategies for different types of cities.

Based on the existing research results, we can find that there are still deficiencies in the following four aspects: (1) In terms of the evaluation index of tourism competitiveness, due to the limitation of county statistical data, previous studies mainly selected five statistical indicators including socioeconomic development, tourism economic development, transportation and location conditions, and tourism resources, and rarely involves three evaluation indexes including market competitiveness, cultural resources competitiveness, environmental influence, and talent competitiveness. (2) The green urbanization system in the new era is more complex and comprehensive, but the index system constructed by existing studies seldom considers the indicators of social progress, urban–rural integration development, and the eco-environment protection indicators are not comprehensive enough. In addition, the research scale is mostly provincial and municipal, and the comprehensive evaluation of green urbanization level at county level is rarely carried out. (3) Existing studies pay little attention to the spatial coupling relationship between tourism competitiveness and green urbanization and the interaction mechanism between them, which seriously hinders the theoretical development of the interaction mechanism between tourism competitiveness and green urbanization, as well as the sustainable development of cities. (4) Existing studies pay less attention to the spatial zoning method of coordination types between tourism competitiveness and green urbanization, as well as the research on the collaborative development path between the two.

The Yellow River Basin (YRB), with its vast area and rich tourism resources, provides the essential conditions for the development of tourism, but the overall development level of tourism is currently low. At the same time, the level of regional economic development and urbanization is lower, and the quality of urbanization is lower, so it is pressing to accelerate the construction of green urbanization. Based on this, it is of vital significance to probe the spatial coupling relationship, interaction mechanism and synergistic development path of county tourism competitiveness and new urbanization on the basis of scientific evaluation of county tourism competitiveness and green urbanization level of counties in the YRB for high-quality urban development and regional sustainable development. Research ideas: (1) Based on multi-source data, build an extended assessment indicator system of county tourism competitiveness and green urbanization level, and then comprehensively evaluate the county tourism competitiveness and green urbanization level in the YRB, and analyze the spatial pattern of county tourism competitiveness and green urbanization and the spatial coupling relationship between them. (2) Based on the conceptual model of the interaction mechanism between tourism competitiveness and green urbanization, the interaction relationship between tourism competitiveness and green urbanization was systematically analyzed. (3) Construct the quantitative division method of the synergistic type areas between tourism competitiveness and green urbanization, and then divide the coordination/discoordination type areas between county tourism competitiveness and green urbanization, and further put forward targeted synergistic development strategies according to different type areas.

## Materials and methods

**Figure 1:**
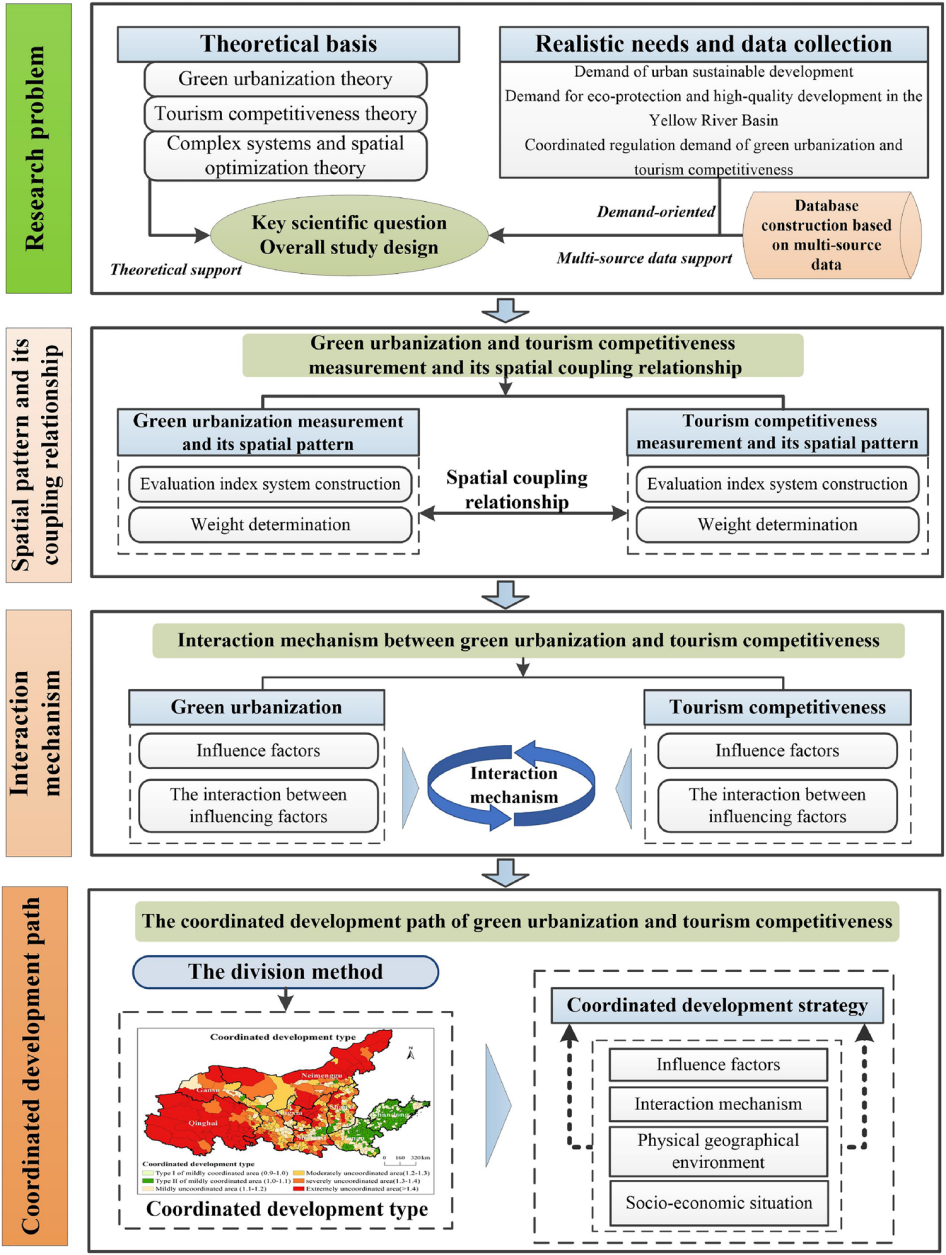
Research framework of this study.

This study was divided into three sections (Fig. 1). The first part was to construct an extended evaluation index system of county tourism competitiveness and green urbanization level based on multi-source data, and then comprehensively evaluate county tourism competitiveness and green urbanization level in the YRB, and analyze the spatial pattern of county tourism competitiveness and green urbanization level and their spatial coupling relationship. In the second part, we systematically analyze the mutual influence between tourism competitiveness and new urbanization, and then explain the interaction mechanism between tourism competitiveness and green urbanization. In the third section, 735 counties are classified according to the quantitative classification method of coordinated development type, and a coordinated development strategy for tourism competitiveness and new urbanization was further proposed for each type area.

### Study area and data sources

**Figure 2:**
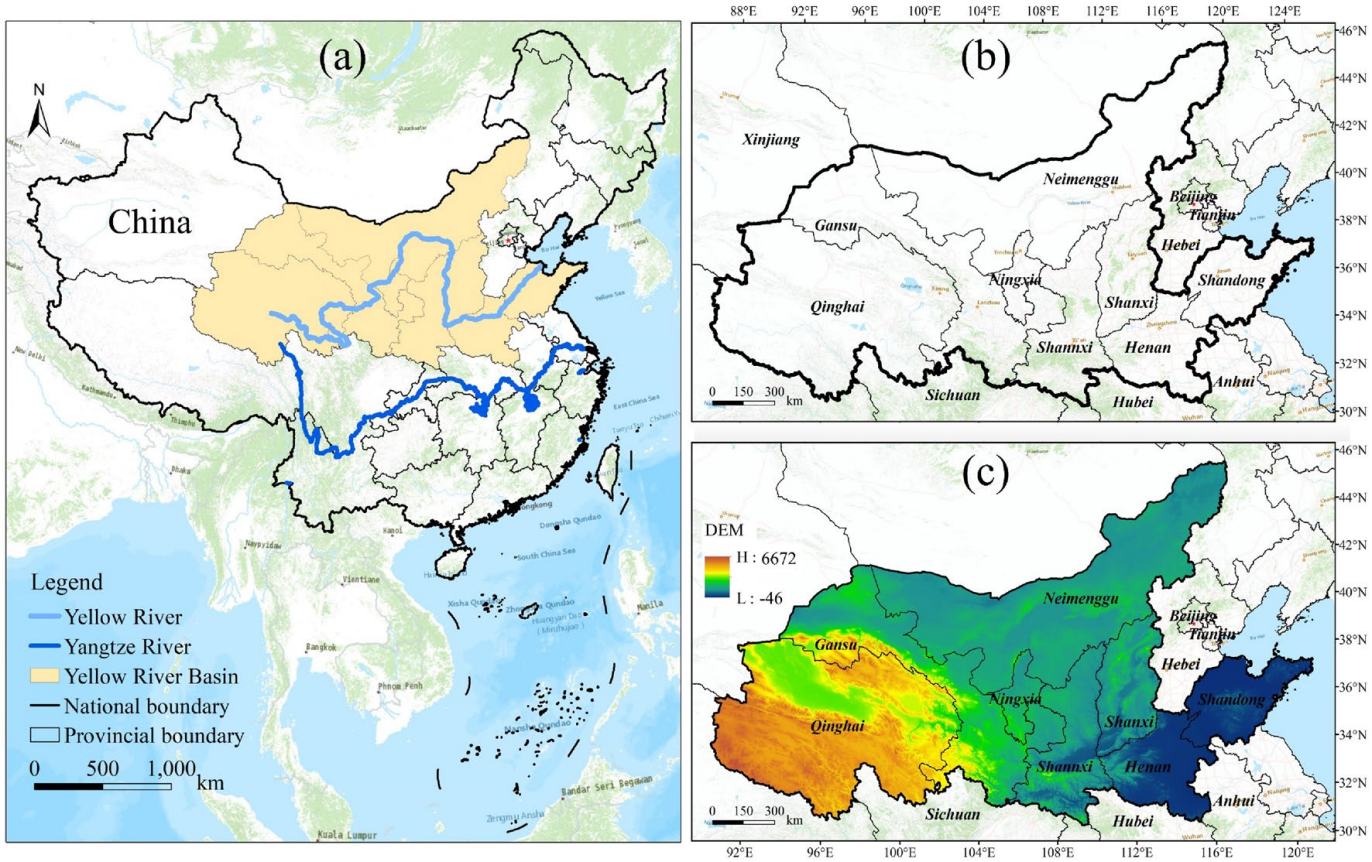
The overview map (**a**), administrative zoning map (**b**), and elevation map of the YRB (**c**), the base map used in the maps in figure number 2, 5, 6, 7, 10 are drawn from the standard map service system of the Ministry of Natural Resources of China (Drawing review No. GS (2019) 1697, http://bzdt.ch.mnr.gov.cn/download.html), and the base map has not been modified. Composed using ESRI ArcGIS 10.2 Software. *This work is licensed under a Creative Commons by Attribution (CC BY 4.0) license. **ESRI, China.

In this study, all counties in the Yellow River Basin (YRB) were taken as the study area (Fig. 2). Referring to the existing studies^32^, the boundaries of the study area are mainly provinces and regions through which the Yellow River flows, including 8 provincial administrative units of Shandong, Henan, Shanxi, Shaanxi, Ningxia, Inner Mongolia (excluding Chifeng, Tongliao, Hinggan League and Hulunbuir), Gansu, and Qinghai Province, and 734 county-level administrative units. The YRB spans three topographic stairways, east and west. The YRB has abundant tourism resources, which provide basic conditions for tourism development, but the overall development degree of tourism is low. Meantime, the level of regional economic development and urbanization level is low, and the development of cities varies widely, so it is urgent to accelerate the construction of green urbanization. In this context, exploring the synergistic relationship, interaction mechanism between green urbanization and tourism competitiveness at counties level in the YRB can effectively promote the regional high-quality and sustainable development.

In this paper, 2022 was taken as the research year. The data types primarily include four aspects: (1) Administrative division data. The regionalization of provincial, municipal and county-level administrative units in the YRB was provided by Resource and Environment Science and Data Center, Chinese Academy of Sciences (RESDC) (https://www.resdc.cn/Default.aspx). (2) POI (Points of Interest) data. The POI data of star scenic spots, star hotels, travel agencies, public toilets and universities in 2022 are provided by RESDC (https://www.resdc.cn/Default.aspx). China's rural tourism key village data in 2022, Chinese history and culture town and village, town of data with Chinese characteristics in 2020, China's national intangible cultural heritage data in 2021, China's key document protection unit data in 2022, and China's traditional villages data in 2022 are come from China's state council web site (https://www.gov.cn/zhengce/zhengceku/). China's time-honored brand data from the Chinese Ministry of Commerce website (http://www.mofcom.gov.cn/article/zwgk/gkgztz/). (3) Remote sensing data. The raster data of land use in 2022 comes from the research results of Yang et al. (2021) (the data has been updated to 2022), with a data resolution of 30 m^33^. PM2.5 concentration raster data in 2022 set was derived from Atmospheric Composition Analysis at Saint Louis University in Beijing, USA Group (https://sites.wustl.edu/acag/datasets/surface-pm2-5/) (the data has been updated to 2022), the spatial resolution of 1 km^34,35^. (4) Socioeconomic statistics data at county level. The county socio-economic statistics data from the county statistical almanac of China and the county socio-economic statistical bulletin.

### The theoretical interaction mechanism of green urbanization and tourism competitiveness

Urban complex ecosystem is a "nature-economy-society" regional composite system integrating natural, economic, and social system^2^. The new urbanization subsystem and the tourism competitiveness subsystem are two open and interrelated subsystems in the urban complex ecosystem. There are complex interaction relations between the two subsystems, which interact and influence each other. The green urbanization adheres to the development concept of "intensive, smart, green and low-carbon", and aims to coordinate urban–rural development, integrate urban and rural areas, improve infrastructure, optimize industrial structure, intensive resources, optimize the ecological environment and make residents livable. Tourism is a comprehensive industry involving "food, accommodation, transportation, travel, shopping and entertainment". The improvement of county tourism competitiveness can promote the integration of various regional resources and industrial integrated development, and play an important role in absorbing urban and rural residents' employment, adjusting industrial structure, driving urban–rural economic development, protecting resources and environment, and improving residents' quality of life. Therefore, green urbanization and tourism competitiveness have a lot in common in terms of development concepts, development goals, focus points and goals, such as urban–rural integration, industrial coordination, resource intensification, ecological environment optimization and inhabitability. On the one hand, the improvement of tourism competitiveness can provide continuous driving force for economic urbanization, industrial low-carbon transformation, social service level, infrastructure construction, ecological environmental protection, etc., and then promote the steady development of new urbanization. On the other hand, the development of green urbanization can improve the quality of economic development, the quality of ecological environment, the perfection of infrastructure, the level of social services, and the level of coordinated development between urban and rural areas, thus improving the development level of tourism service industry, the competitiveness of infrastructure, market, service, resource, talent and environmental influence, and accelerating the virtuous circle within the tourism industry.

**Figure 3:**
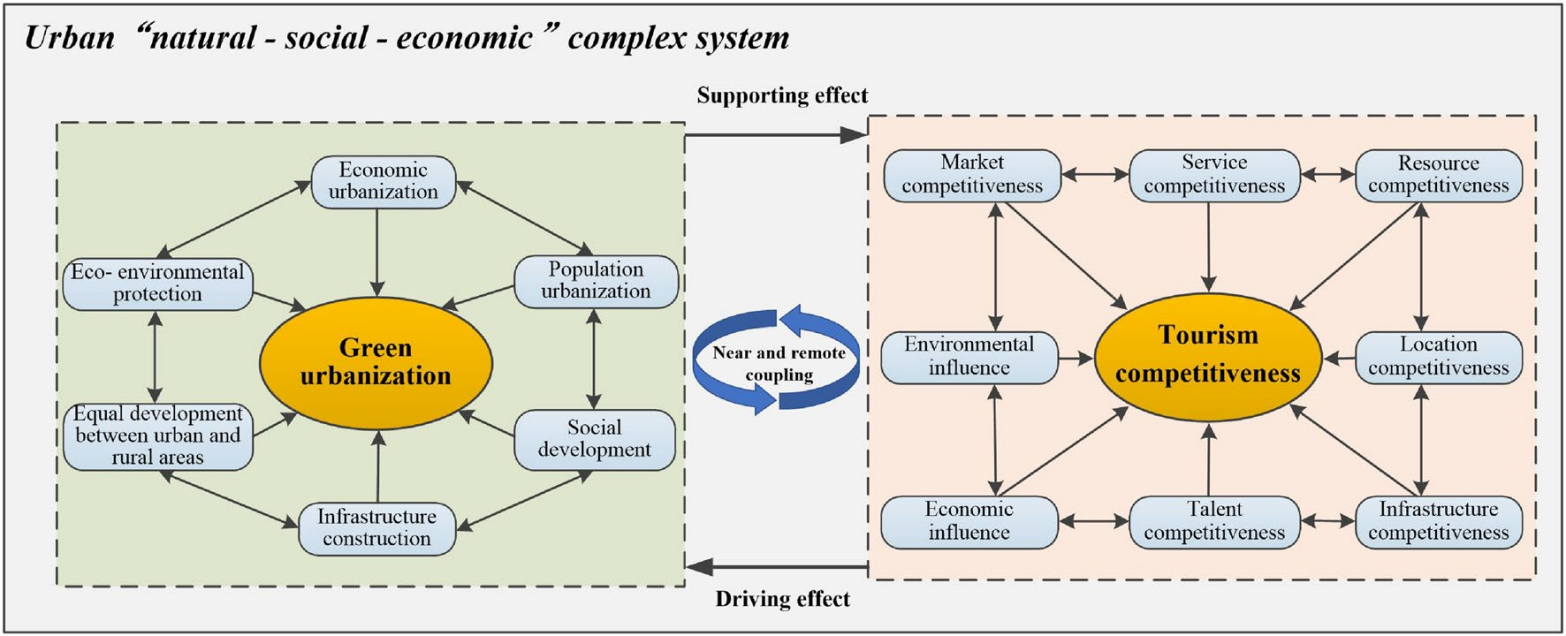
The theoretical interaction mechanism of green urbanization and tourism competitiveness.

Based on this, we put forward the theoretical mechanism of green urbanization and tourism competitiveness (Fig. 3), and put forward the following hypotheses: (1) The development of green urbanization has a positive supporting effect on tourism competitiveness. (2) Tourism competitiveness plays a positive role in promoting green urbanization.

### Quantitative evaluation of green urbanization level

Green urbanization is a coordinated urbanization of industry, pop, land, society, and environment. It is oriented towards improving quality, centered on the people, driven by technological innovation, and based on the principles of green, low-carbon, and overall planning^4,7,8^. The green urbanization has great differences compared with traditional urbanization, which are reflected in the following ways: First, green urbanization pays more attention to the economic development quality and urbanization construction, as well as ecological and environmental protection^12,19,20^. Second, the green urbanization is based on urban–rural integration, industrial interaction, resource saving, pleasant living environment as the elementary features of urbanization^7–10^.

On the basis of a deep understanding of the connotation and essence of green urbanization, we have established a evaluation index system of green urbanization level composed of 6 criteria layers (18 specific indicators) including quality of economic development, population development, social progress, infrastructure construction, urban–rural integrated development, and eco-environmental protection (Table 1). In view of the difference of positive and negative properties of evaluation indicators, this study selects the method of maximum value standardization to standardize the indicator data. The entropy method was used to calculate the indicator weight.

**Table 1:** Evaluation index system of green urbanization.

Destination	Criteria	Indicators
Green urbanization level	Quality of economic development	Per capita GDP
		Proportion of secondary and tertiary industries in GDP
		Per capita savings balance of urban and rural residents
		Per capita fixed asset investment
	Population development	Urbanization rate
		Proportion of employed population in secondary and tertiary industries
	Social progress	Number of primary and secondary schools per 10,000 people
		The number of teachers per 10,000 people
		Per capita Total retail sales of consumer Goods
	Infrastructure construction	Density of way network
		Density of railway network
		Number of stadiums and gymnasiums per 10,000 people
		Number of public library books per 10,000 people
		Number of beds in hospitals and health centers per 10,000 people
	Equal development between urban and rural areas	Per capita disposable income ratio of urban and rural residents
	Eco-environmental protection	Forest coverage rate
		Grassland coverage rate
		PM2.5 concentration

### Quantitative evaluation of tourism competitiveness

County tourism is a tourism area with regional characteristics and complete functions based on the geographical space of county administrative districts and the participation of county governments, tourism departments and enterprises. It is supported by local tourism resources with local characteristics, guided by the market and centered on tourism products^17^. Combined with the previous study results^6,16–19^, this study believes that the county tourism competitiveness is the past, present and future tourism market position and competitiveness level created by the county government, tourism departments and enterprises to realize the sustainable development of its own tourism destination and tourism industry by utilizing the local resource advantages and various opportunities.

On the basis of a deep understanding of the concept and connotation of county tourism competitiveness, this study follows the principles of concise and scientific, systematic, targeted and regional combination, the availability of index data and so on, and carries out the selection of specific evaluation indicators of county tourism competitiveness and construction of evaluation index system. Finally, the evaluation index system of county tourism comprehensive competitiveness is constructed, which consists of 3 criteria layers, 8 subdivision criteria layers and 23 specific indicators (Table 2). Tourism competitiveness of a county is a comprehensive evaluation of the overall strength of tourism in a specific period, reflecting the past, present and future tourism market position and competitiveness level of a county, which is mainly reflected in three aspects: real tourism competitiveness, potential tourism competitiveness and tourism competitive influence ^6,16,19,20^. Among them, the real competitiveness of tourism is reflected by 7 specific indicators in 2 aspects, including market competitiveness and service competitiveness. The potential competitiveness of tourism is reflected by 10 specific indicators in four aspects: cultural resource competitiveness, location competitiveness, infrastructure competitiveness and human competitiveness. The competitive influence of tourism is reflected by six specific indicators in two aspects, namely economic influence and environmental influence. For the different positive and negative orientations of the indicators in the evaluation index system, this paper selects the maximum difference dormalization method to standardize the index data. The entropy method was used to calculate the indicator weight.

**Table 2:** Comprehensive evaluation index system of tourism competitiveness.

Destination	Criteria	Elements	Indicators
Tourism competitiveness	Tourism realistic competitiveness	Market competitiveness	Number of A-5A tourist attractions
			Number of key towns and villages for rural tourism
			The number of historical and cultural cities, towns and villages
			Number of characteristic towns
		Service competitiveness	Number of star-rated hotels
			Number of travel agencies
			Number of public toilets
	Tourism potential competitiveness	Resource competitiveness	Number of national intangible cultural heritage
			Number of national key cultural protection units
			Number of traditional villages
			Number of Chinese time-honored brands
		Location competitiveness	Distance from the central city
		Infrastructure competitiveness	Distance from high speed rail station
			Distance from airport site
			The density of the way network
		Talent competitiveness	Number of colleges and universities
			The proportion of employees in the tertiary industry
	Tourism competitive influence	Economic influence	Per capita GDP
			Proportion of output value of tertiary industry
			Proportion of total fixed asset investment in GDP
		Environmental influence	Forest coverage rate
			Grassland coverage rate
			PM2.5 concentration

### Bivariate spatial autocorrelation method

We adopt the *Bivariate spatial autocorrelation (BSA)* method to analyze whether there is a prominent spatial dependence between green urbanization and tourism competitiveness ^36^. The equation is:

1 $${I}_{\text{ur}}=\frac{n\sum_{i}^{n}\sum_{j\ne 1}^{n}{w}_{ij}{z}_{i}^{u}{z}_{j}^{r}}{(\text{n}-1)\sum_{i}^{n}\sum_{j\ne 1}^{n}{w}_{ij}}$$

where ${I}_{\text{ur}}$ is the global bivariable Moran's *I* index between green urbanization and tourism competitiveness. *n* is the count of units, ${z}_{i}^{u}$ is the green urbanization level of unit *i* adjacent to unit *j*, ${z}_{j}^{r}$ is the tourism competitiveness of unit *j* adjacent to unit* i*, ${w}_{ij}$ is the weight matrix.

### Optimal parameters-based geographical detector model

The Optimal parameters-based geographical detector (OPGD) model is an improvement model based on Geographical Detector (GD) model^37,38^, which can explore the potential factors or explanatory variables from the perspective of space, and explore the potential interaction of variables^38^. The biggest advantage of OPGD model compared with the traditional GD model is that it can identify the optimal method of data discrete and the optimal number of breakpoints.

(1) Factor detector. The detector can analyze the influencing factors of green urbanization and tourism competitiveness. The model formula:

2 $$q=1-\left[\frac{\sum_{h=1}^{L}\sum_{i=1}^{{N}_{h}}{\left({Y}_{hi}-\overline{{Y }_{h}}\right)}^{2}}{\sum_{i=1}^{N}{\left({Y}_{i}-\overline{Y }\right)}^{2}}\right]=1-\frac{\sum_{h=1}^{L}{N}_{h}{{\sigma }_{h}}^{2}}{N{\sigma }^{2}}$$

In the formula, *q* value is the explanatory power of factor. *h* and *N*_*h*_ is the count of layers and samples, ${\sigma }_{h}$ is the variance of the green urbanization and tourism competitiveness. ${Y}_{i}$ is the level of green urbanization and tourism competitiveness.

**Figure 4:**
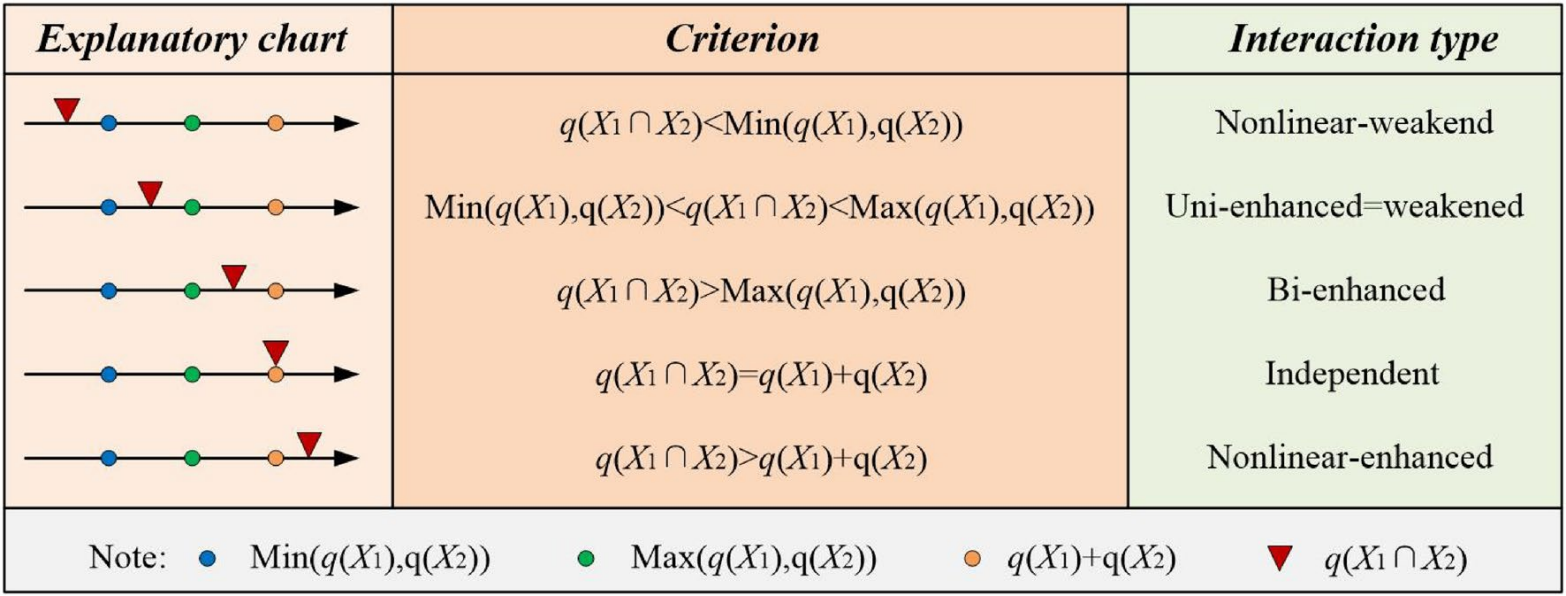
Interaction types.

(2) Interaction detector. The detector can identify the interaction between influencing factors (the comprehensive effect of evaluation factors on green urbanization and tourism competitiveness). The five types of interaction are illustrated in Fig. 4.

### The division method of coordination type between green urbanization and tourism competitiveness

In order to quantitatively divide the coordination types between green urbanization and tourism competitiveness, and further propose targeted collaborative development strategies according to different types of areas, this study puts forward the quantitative division method of coordination type. The detailed steps of the division method are as follows: Firstly, the extreme value standardization method is adopted to standardize the tourism competitiveness value and the green urbanization level respectively. Secondly, the ratio between the standardized value of green urbanization and the standardized value of tourism competitiveness is calculated. Finally, according to the ratio and interval value of the two, the collaborative type was divided. The formula is:

3 $${X}_{i}=\frac{{x}_{i}}{{Max(x}_{i})}$$

4 $${Y}_{i}=\frac{{y}_{i}}{{Max(y}_{i})}$$

5 $${A}_{i}=\frac{{X}_{i}}{{Y}_{i}}$$

6 $${B}_{i}=\left\{\begin{array}{c} Type I of mildly coordinated area , 0.9{<A}_{i}<1.0 \\ Type II of mildly coordinated area , 1.0{<A}_{i}\le 1.1 \\ Mildly uncoordinated area , 1.1{<A}_{i}\le 1.2 \\ Moderately uncoordinated area, 1.2{<A}_{i}\le 1.3 \\ severely uncoordinated area , 1.3{<A}_{i}\le 1.4 \\ Extremely uncoordinated area , 1.4{<A}_{i}\end{array}\right.$$

where ${x}_{i}$ and ${y}_{i}$ is the green urbanization and tourism competitiveness. ${Max(x}_{i})$ and ${Max(y}_{i})$ is the maximum value of green urbanization and tourism competitiveness. ${X}_{i}$ and ${Y}_{i}$ is the standardized value of the green urbanization and tourism competitiveness. ${A}_{i}$ is the ratio of ${X}_{i}$ to ${Y}_{i}$, ${B}_{i}$ is the coordinated development type.

## Results and discussion

### The spatial coupling relationship between green urbanization and tourism competitiveness

#### Spatial pattern of green urbanization level

**Figure 5:**
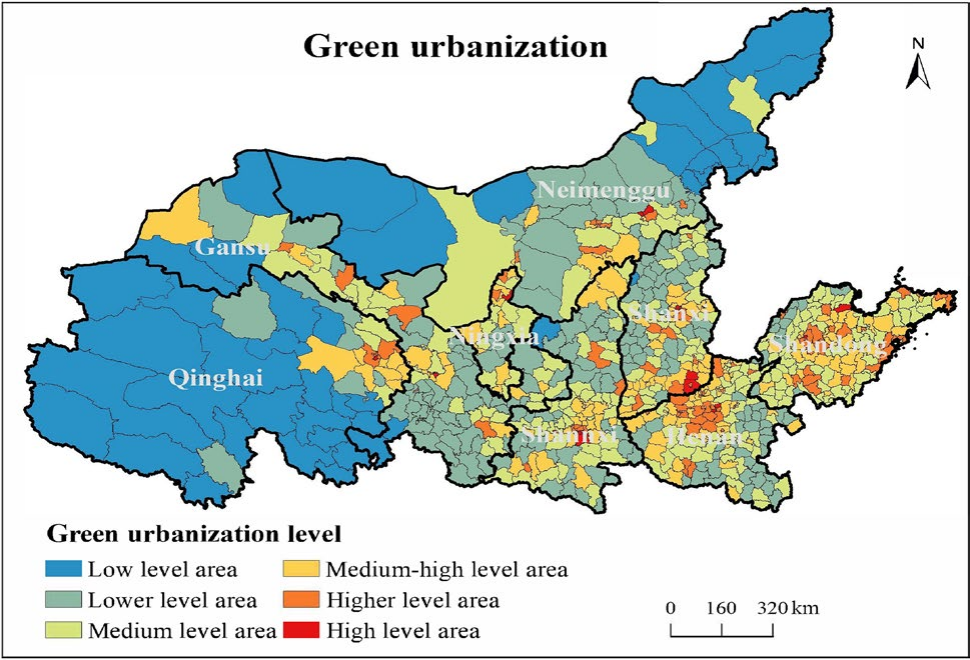
Spatial distribution of green urbanization in the YRB.

This paper selects Jenks natural break point method to grade the green urbanization level, which can be divided into six types: low level area, lower level area, medium level area, medium–high level area, higher level area, and high level area (Fig. 5).

As shown in Fig. 5, the green urbanization level show a multi-core circle structure, with provincial capitals, prefecture-level city districts and economically developed counties as high-value centers, and the trend of concentrated distribution is significant. In detail, the low-level areas are mainly distributed in the central and western regions of Qinghai Province, the northwestern regions of Gansu Province, and the western and northeastern regions of Inner Mongolia. The lower-level areas are mainly distributed in the central and southern parts of Gansu, the central parts of Ningxia, the central parts of Inner Mongolia, the central and northern parts of Shaanxi Province, the northern parts of Shanxi Province, the counties under the jurisdiction of Shangqiu and Zhoukou City in eastern Henan, the southeastern counties under the jurisdiction of Nanyang City in southern Henan and the counties under the jurisdiction of Zhumadian City. The higher and high level areas are mainly distributed in the counties and districts under the jurisdiction of the provincial capital Xining City, the Chengguan District under the jurisdiction of the provincial capital Lanzhou City, the economically developed counties and districts of Gansu Province (Jiayuguan, Ganzhou, Liangzhou, Qinchuan), the provincial capitals Yinchuan City, the provincial capitals Xi 'an City, Baota District, the provincial capitals Taiyuan City, Jincheng, and the provincial capitals Zhengzhou has jurisdiction over counties, economically developed counties in the west and north of Henan Province (Jiyuan, Luoyang city jurisdiction, Nanyang city jurisdiction, Puyang city jurisdiction), the provincial capital of Jinan City, the southeast coast of Shandong Province. The medium and medium–high level areas are mainly distributed around the high and higher level areas, showing an obvious circular distribution structure.

#### Spatial pattern of tourism competitiveness

This paper selects Jenks natural break point method to grade the calculation results of county tourism competitiveness index, which can be divided into six types: low competitiveness area, lower competitiveness area, medium competitiveness area, medium–high competitiveness area, higher competitiveness area, and high competitiveness area.

**Figure 6:**
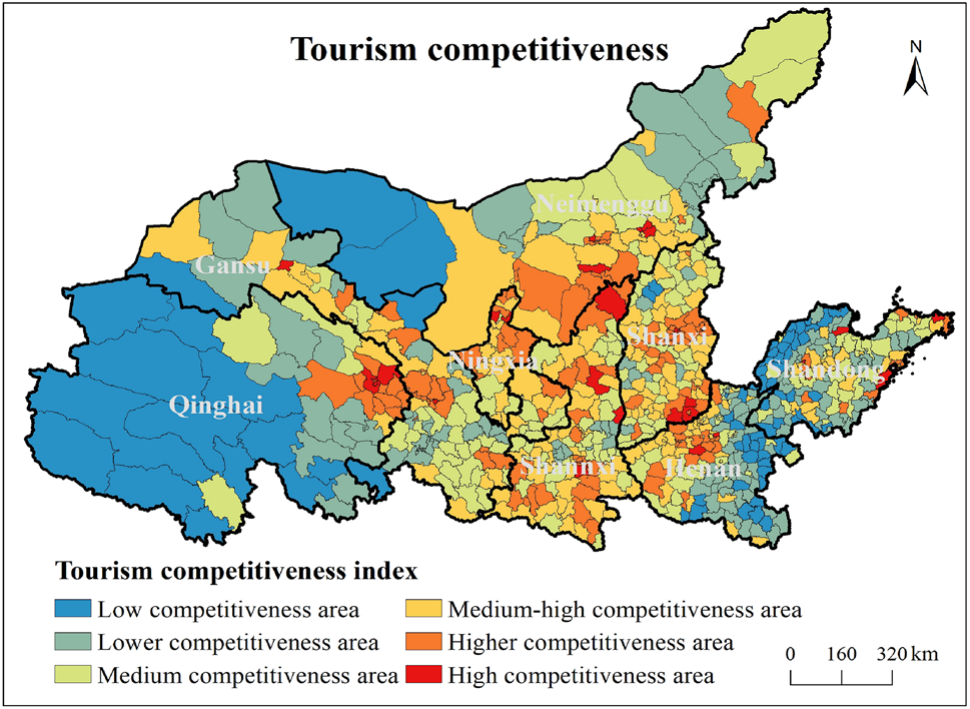
Spatial distribution of county tourism competitiveness in the Yellow River Basin.

As shown in Fig. 6, the tourism competitiveness of counties in the Yellow River Basin shows a polycentric spatial differentiation. In detail, the regions with low and lower competitiveness are mainly distributed in western Qinghai Province, western Inner Mongolia, Northern Shaanxi Plateau, northern Shanxi, northern Henan, eastern Henan, southern Henan, western and northern Shandong. The medium and high competitiveness areas are mainly distributed in northern and southern Gansu, northern and eastern Inner Mongolia, northern Shanxi, central Henan, southern Henan and central Shandong. High and medium–high competitiveness areas are mainly distributed in eastern Qinghai Province, northern and central Gansu, northern Ningxia, northern Shaanxi and southern Shaanxi Province, central Shanxi, southern Shanxi, western Henan Province, central Shandong Province, and southeast coastal areas of Shandong Province.

#### Spatial coupling pattern between green urbanization and tourism competitiveness

The results of model analysis show that the global bivariate Moran's *I* index between tourism competitiveness and green urbanization variables is 0.352, indicating that there is a significant positive spatial agglomeration relationship between tourism competitiveness and green urbanization variables, that is, they are in the phase of benign resonant coupling.

**Figure 7:**
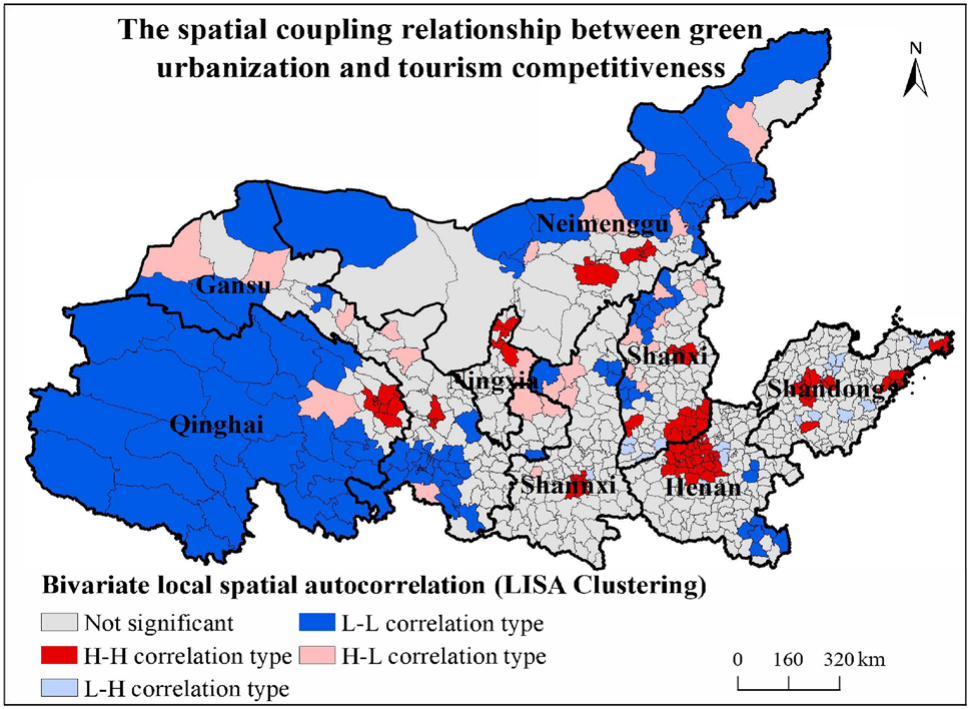
Spatial coupling pattern between green urbanization and tourism competitiveness in the YRB.

The global bivariate Moran's *I* index can only provide a global assessment of the spatial correlation between tourism competitiveness and green urbanization as a whole, but it suffers from the drawback that it ignores the instability of spatial process and cannot judge the local spatial agglomeration characteristics. Therefore, with the help of local bivariate spatial autocorrelations, we analyzed the local spatial agglomeration characteristics of county-scale tourism competitiveness and green urbanization in the Yellow River Basin. As shown in Fig. 7, LISA agglomeration map contains four types of local spatial agglomeration, namely high-high type (HH), low–high type (LH), low-low type (LL) and high-low type (HL). Overall, there is a significant spatial agglomeration between the tourism competitiveness and new urbanization in the Yellow River Basin, and the spatial agglomeration types are mainly HH and LL types. In detail:

High-value agglomeration areas (H–H correlation type) indicate that tourism competitiveness and green urbanization are high. This type of district is mainly distributed in the provincial capital and its surrounding areas (Xining City, Lanzhou City, Yinchuan City, Hohhot city, Xi 'an City circle, Taiyuan City circle, Zhengzhou City circle, Jinan City Circle), Ordos City, Jincheng City, Luoyang City, Jiyuan City, Jiaozuo City, Jining City, Zibo City, Qingdao City, Weihai City. The reason is that the high-value agglomeration area of provincial capital is located in the political, economic and cultural center, with strong economic strength, relatively perfect urban infrastructure and social security system, high ecological environment quality and high level of green urbanization. At the same time, the historical and cultural accumulation is profound, tourism resources are rich, and tourism competitiveness is also high. Therefore, its green urbanization level and tourism competitiveness show a high value coupling development state. Ordos in Inner Mongolia has benefited from the economic development dividends brought by the development of mining industry and animal husbandry, with relatively perfect social services and urban infrastructure, and a high level of green urbanization construction. On the other hand, Ordos Plateau is rich in eco-tourism resources and has strong tourism competitiveness. Therefore, its green urbanization level and tourism competitiveness show a high value coupling development state. Luoyang, Zibo,and Jining City are all tourist cities with a long history and profound culture, with rich historical and cultural heritage and ecological tourism resources; At the same time, it has a solid industrial foundation, strong economic strength, perfect urban infrastructure construction, high social welfare level, and remarkable achievements in green urbanization. Therefore, its green urbanization level and tourism competitiveness show a high value coupling development state. Jincheng, Jiyuan, and Jiaozuo City are located in the south of Taihang Mountain, are national forest cities and national garden cities, rich in eco-tourism resources; At the same time, its economic strength is strong, the urban infrastructure and social security system is relatively perfect, the urban–rural income gap is small, the living environment is suitable, and the green urbanization construction has achieved remarkable results. Therefore, its green urbanization level and tourism competitiveness show a high value coupling development state. The development of coastal eco-tourism in Qingdao and Weihai started earlier, and the level of tourism competitiveness has always been in an advantageous position. At the same time, its industrial structure is perfect, the economic strength is strong, the urban infrastructure is perfect, the ecological environment is beautiful, so the green urbanization level is high.

Low-value cluster (L–L correlation type) indicates that tourism competitiveness and green urbanization level are low. This type of district is mainly distributed in central and western counties of Qinghai Province, southwestern counties of Gansu Province, western and eastern regions of Inner Mongolia (Alashan City), Yulin City of Shaanxi Province (Dingbian County, Zizhou City, etc.), Qinzhou City and Shuozhou City of Shanxi Province, western counties of Shangqiu City (Suixian County and Taikang County) of Henan Province, Zhumadian City and eastern counties of Xinyang City (Xincai County, Zhengyang County, Xixian County, etc.). Among them, the counties in the central and western parts of Qinghai Province and the counties in the southwest of Gansu Province are located in the plateau areas that are rarely visited by people, with low economic development and slow urbanization development. Green urbanization is still in its infancy. Meanwhile, eco-tourism resources are rich, but the level of tourism resources development is low, and tourism infrastructure is relatively backward. Therefore, tourism competitiveness and green urbanization level are in a low level coupling development stage. Alashan and Yulin City are located in the desert areas with poor ecological environment. The urbanization level is low. The primary and secondary industries are the main industries, and the development gap between urban and rural areas is large. At the same time, tourism resources are scarce and tourism infrastructure is relatively backward. Therefore, tourism competitiveness and green urbanization level are in a low level coupling development stage. Qinzhou City, Shuozhou City, western counties of Shangqiu City, and eastern counties of Xinyang and Zhumadian City are all agricultural counties. The industrial structure is dominated by the primary and secondary industries, the economic development is relatively extensive, the tourism resources are relatively scarce, and the tourism infrastructure is not perfect. Therefore, the level of green urbanization and the level of green urbanization are at a low level coupling development stage.

Low-value heterogeneous areas (Low–High correlation type) indicate that tourism competitiveness is low, while the level of green urbanization is high. This type of area is mainly distributed in the central and eastern provinces of the Yellow River basin, including Lintong District of Xi 'an, southern counties of Yuncheng (Wanrong County, Linyi County, Hengqu County, etc.), Zhongmu County of Zhengzhou City, Xinxiang Yuanyang County, central counties of Shandong Province (Guarao County, Boxing County, Hengtai County, Wulian County, etc.), and eastern counties of Shandong Province (Zouping District, Futian District). Among them, the counties around the core cities, such as Lintong District of Xi 'an, Zhongmou County of Zhengzhou, and Yantai District of Shandong Province (Zouping District and Futian District), are positively affected by the economic radiation of the core cities, with fast economic development, perfect infrastructure and high level of green urbanization. However, as an economic development zone, the resource endowment is poor, the tourism resources are scarce, the tourism infrastructure is imperfect, and the tourism market demand is low, so the tourism competitiveness is low. The development of tourism competitiveness in the southern part of Yuncheng City, the middle part of Shandong Province, and Yuanyang County are mainly restricted by the factors of resource endowment, location condition and tourism market. However, in the process of rapid urbanization, the infrastructure and social security system are gradually improved, the ecological environment is gradually improved, and the level of green urbanization has been submitted.

The high value heterogeneous area (High-Low correlation type) indicates that the tourism competitiveness is high but the new-type urbanization level is low. This type of area is mainly distributed in the western tourist cities of Gansu Province (Dunhuang City, Yumen City, etc.), the central and eastern tourist cities of Inner Mongolia (Erlianhot City, Xilinhot City, etc.), Yulin City of Shaanxi Province (Jingbian County, Wuqi County, Zhidan County, etc.), and the counties of Luliangshan Mountain region of Shaanxi Province (Pinglu District, Hunyuan County, Ningwu County, Xing County, Loufan County, Liulin County and Jiaokou County). Among them, the western cities of Gansu Province have a large number of historical and cultural monuments and ancient buildings, and the development of heritage tourism is good and the tourism competitiveness is strong, but the development level of green urbanization is low. The central and eastern Inner Mongolia cities mainly develop grassland eco-tourism, which has strong tourism competitiveness, but the development level of green urbanization is low. Yulin City in Shaanxi province mainly develops desert tourism and has strong tourism competitiveness, but its economic development mainly focuses on high-polluting industries such as coal mining and smelting industry, and its ecological environment is poor, and the development level of green urbanization is low. In recent years, the county area of Luliang Mountain in Shaanxi Province develops eco-tourism around eco-tourism resources, and the tourism competitiveness is gradually enhanced. However, the overall development speed of green urbanization is slower than the competitiveness of tourism.

### Interactive mechanism between green urbanization and tourism competitiveness

#### The influence of green urbanization on tourism competitiveness

(1) Analysis results of factor detector

The analysis results of OPGD model show (Table 3) that the explanatory power (q value) of green urbanization, quality of economic development, population development, social progress, infrastructure construction, urban–rural integrated development, and eco-environmental protection factors on tourism competitiveness is greater than 0.1, and Pearson correlation coefficient is positive, and all pass the 1% significance level test. It shows that the above factors have a significant positive impact on tourism competitiveness. Among them, the *X1* has the greatest impact on tourism competitiveness, followed by the *X3*, *X4*, *X5*, *X2, X7,* and *X6*. The reason for this, the development of population provides population support for urban development and construction and tourism-related service industry, and directly promotes the development of economic and tourism-related service industry, and quality of economic development. Quality of economic development provides strong financial support for social development and infrastructure construction and indirectly promotes the comprehensive competitiveness of tourism. Social progress and infrastructure construction provide perfect infrastructure and comfortable tourism experience for tourism development, which directly promotes the tourism competitiveness. Eco-environmental protection factors have many influences on the tourism competitiveness. Firstly, the regions with higher ecological environmental quality generally have better tourism resource endowment. Secondly, the improvement of eco-environment quality can promote the development of regional eco-tourism. In addition, tourists' travel experience and impression can be improved by improving the quality of eco-environment, and the rate of repeat visits can be increased. The factor of urban–rural integrated development has the least impact on the tourism competitiveness.

**Table 3:** The impacts of green urbanization and its subsystems on tourism competitiveness.

Number	Influencing factor	*q* value	Pearson correlation coefficient
X1	Green urbanization level	0.546***	+0.743***
X2	Quality of economic development	0.204***	+0.451***
X3	Population development	0.383***	+0.536***
X4	Social progress	0.368***	+0.593***
X5	Infrastructure construction	0.312***	+0.507***
X6	Urban–rural integrated development	0.129***	+0.270***
X7	Ecological environmental protection	0.171***	+0.398***

**Indicates 5% significance level; ***indicates 1% significance level.

(2) Analysis of interaction between factors

**Figure 8:**
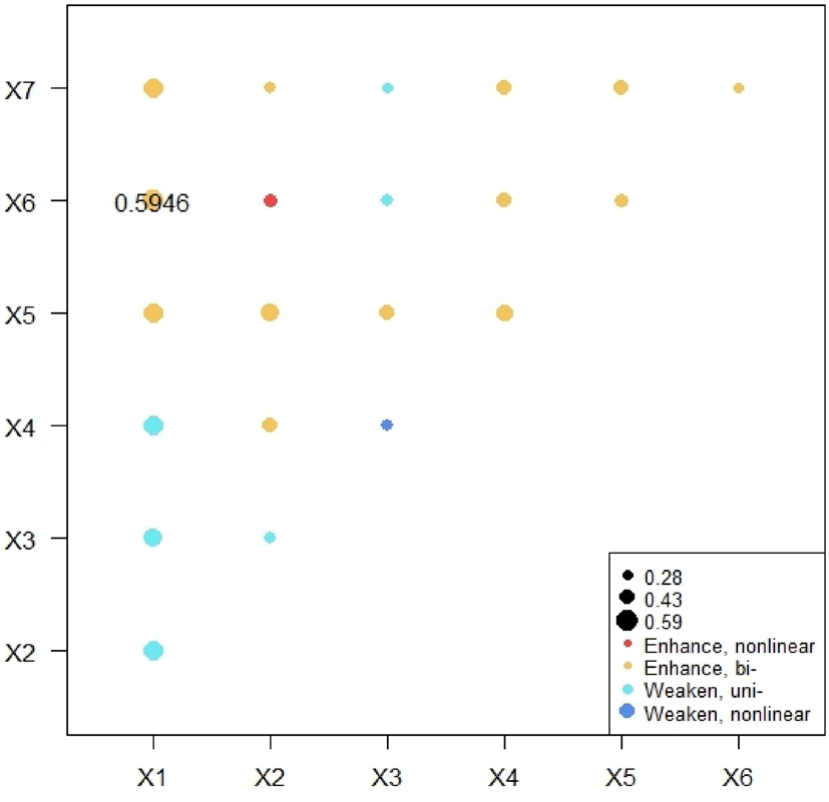
Interactive influence of influencing factors on tourism competitiveness.

The results are shown in Fig. 8, the interaction types of factors include double-factor enhancement, nonlinear enhancement and attenuation. In detail, the interaction between economic development quality and population development, social progress, infrastructure construction, urban–rural coordinated development, and eco-environmental protection has a strong interaction effect on tourism competitiveness. The interaction between population urbanization, infrastructure construction level and urban–rural integration level has a strong interactive impact on the tourism competitiveness, but the interaction type of population development, social progress, and eco-environmental protection is weaker type. The interaction between social progress, infrastructure construction, urban–rural coordinated development, and eco-environmental protection has a strong interaction effect on the tourism competitiveness, and the interaction between urban–rural coordinated and eco-environmental protection has a strong interaction effect on the tourism competitiveness. It shows that there is a close link between social progress, infrastructure construction, urban–rural coordinated development, and eco-environmental protection.

#### The influence of tourism competitiveness on green urbanization

(1) Analysis results of factor detector

The results show (Table 4) that the explanatory power (q value) of tourism competitiveness, market competitiveness, service competitiveness, resource competitiveness, location competitiveness, infrastructure competitiveness, talent competitiveness and environmental influence factors are all greater than 0.1, and Pearson correlation coefficient is positive, and all pass the 1% significance level test. All the above eight factors have a significant positive impact on the level of green urbanization. The X6 has the greatest influence on the green urbanization level, followed by X1, X3, X5, X4, X2, X9, and X7. It shows that tourism competitiveness and its subsystems can observably promote the green urbanization. The reason is that the construction of transportation infrastructure such as high-speed rail, highway and bus (infrastructure competitiveness) and the construction of tourism service facilities such as star-rated hotels, travel agencies and public toilets (service competitiveness) can directly promote the improvement of the green urbanization. Superior geographical location and talent reserve in the service industry significantly promote the improvement of regional competitiveness and talent competitiveness, and also serve as a continuous driving force to promote the green urbanization. Market competitiveness, resource competitiveness and environmental influence are not only important resource endowment and basic factors required by tourism development, but also core factors of green urbanization development. Economic impact factors have the least influence on the green urbanization. The reason is that compared with the traditional urbanization that unilaterally pursues spatial expansion and economic size, green urbanization pays more attention to the comprehensive, balanced and green development of social development potential (education, science and technology, etc.), infrastructure construction, eco-environmental protection, urban and rural balanced development and other aspects.

**Table 4:** The impact of tourism competitiveness and its subsystems on green urbanization.

Number	Influencing factor	*q* value	Pearson correlation coefficient
X1	Tourism competitiveness level	0.561***	+0.743***
X2	Market competitiveness	0.279***	+0.487***
X3	Service competitiveness	0.496***	+0.706***
X4	Resource competitiveness	0.303***	+0.541***
X5	Location competitiveness	0.487***	+0.641***
X6	Infrastructure competitiveness	0.597***	+0.774***
X7	Talent competitiveness	0.181***	+0.417***
X8	Economic influence	0.051***	+0.061
X9	Environmental influence	0.248***	+0.382***

**Indicates 5% significance level; *** indicates 1% significance level.

(2) Analysis of interaction between factors

**Figure 9:**
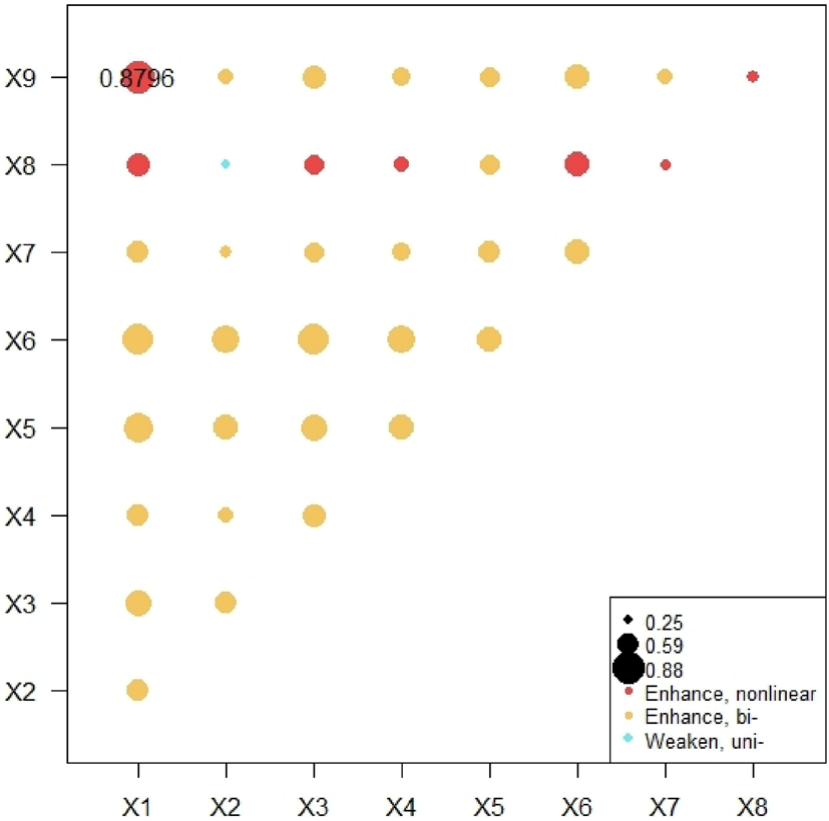
Interactive influence of influencing factors on green urbanization.

As shown in Fig. 9, the tourism competitiveness and environmental influence factors have the largest interactive influence on the green urbanization (q value = 0.879). The second is the interactive influence of tourism comprehensive competitiveness and service competitiveness, location competitiveness and infrastructure competitiveness, location competitiveness and market competitiveness, service competitiveness and resource competitiveness. The interactive influence of infrastructure competitiveness, market competitiveness, service competitiveness, resource competitiveness, location competitiveness, talent competitiveness, economic influence, and environmental influence. The combined effect of market competitiveness and economic influence did not enhance the interaction effect on the green urbanization. On the whole, the interaction effect of tourism competitiveness factors and their subsystems on the level of new urbanization is significantly higher than that of single factors, indicating that tourism competitiveness and the complex force among its subsystems have an obvious promoting effect on the level of new urbanization.

#### Interactive mechanism between green urbanization and tourism competitiveness

(1) Driving effect of tourism competitiveness on green urbanization. (1) Tourism competitiveness promotes economic urbanization construction. Tourism is a comprehensive industry involving "food, accommodation, transportation, travel, shopping and entertainment". County tourism plays a significant role in absorbing employment, adjusting industrial structure and driving regional economic development. It promotes economic urbanization by promoting the development of local characteristics industries and the transformation of service industries. (2) Tourism competitiveness promotes the equal development between urban and rural areas. At present, China's county cities generally exist dual structure problems such as urban–rural division and large urban–rural gap, and tourism can bring capital flow, people flow and logistics to rural areas by virtue of strong integration and aggregation. With the advent of the age of mass leisure, tourists' demand for the original ecological tourism distributed in the countryside is getting higher and higher. In remote rural areas with rich tourism resources, rich cultural deposits and rich ethnic customs, tourism can integrate various tourism resources and funds, combine tourism development with poverty alleviation, improve farmers' income, and promote the equal development between urban and rural areas. (3) The promotion of tourism competitiveness can promote population urbanization. As a labor-intensive industry, tourism can not only provide direct employment, but also promote indirect employment. On the one hand, it can provide a large number of employment opportunities for travel agencies, catering and accommodation, entertainment and leisure, tourist attractions and other directly related industries. On the other hand, it can also provide employment opportunities for transportation, health care, insurance and other industries indirectly related to tourism, increase the employment rate of urban and rural population, and ultimately promote population urbanization. (4) County tourism competitiveness promotes infrastructure construction and social comprehensive development. On the one hand, cities with strong tourism competitiveness will carry out tourism-related infrastructure construction and public service supporting facilities to optimize tourism services and increase tourists' satisfaction. On the other hand, the improvement of tourism competitiveness will attract a large number of financial funds and social funds into the development of tourism and the construction of related supporting industries, thus promoting the construction of infrastructure and improving the social services. (5) Tourism competitiveness promotes ecological urbanization. Tourism development can promote the construction of more parks and green landscapes in cities and towns, improve the ecological environment and air quality. In addition, the improvement of tourism competitiveness and tourism innovation level is conducive to the emergence of new tourism business forms, promote the continuous transformation of urban functions and industrial structure to the direction of green, ecological and low-carbon, and promote ecological urbanization.

In summary, the improvement of tourism competitiveness can provide continuous driving force for economic urbanization, industrial transformation and upgrading, population urbanization, social services, infrastructure construction, cultural inheritance, ecological and environmental protection, and thus promote the steady development of green urbanization.

(2) The supporting effect of green urbanization on tourism competitiveness. Population agglomeration brings a large number of labor resources to cities and towns, and also provides population support for urban development and construction and tourism-related service industry, which directly promotes the development of economic and tourism-related service industry. Economic development provides strong financial support for social progress and infrastructure construction and indirectly promotes the tourism competitiveness. Eco-environment protection can promote the development of regional eco-tourism, and at the same time, it can also improve the tourism experience and impression of tourists by improving the quality of ecological environment, and increase the repeat rate. Along with the construction and development of green urbanization, with its excellent conditions such as perfect infrastructure, environment and modern education, green urbanization has enhanced the attraction of scientific and technological innovative talents. At the same time, green urbanization has a "siphon effect", enabling innovative elements such as capital, technology and knowledge to gather in cities and towns, providing important support for the development of tourism industry. On the whole, the development of green urbanization can support the development of tourism service industry, tourism infrastructure construction, tourism personnel training and eco-environment improvement, so as to enhance the development level of tourism service industry, tourism infrastructure competitiveness, talent competitiveness and environmental influence. In addition, in the process of new urbanization development, tourism industry agglomeration will be formed, and high-tech innovative tourism enterprises will constantly impact traditional tourism enterprises, enhance the competition among tourism industries and improve the competitiveness of tourism products. The formation of "locking effect" is helpful to improve the science and technology level, and further promote the improvement of tourism innovation level. When the tourism industry enters the stage of rapid development and a virtuous cycle, it will attract a large number of financial funds and social funds into the tourism development and the construction of related supporting industries, drive the local economic influence, market competitiveness, service competitiveness and resource competitiveness, and accelerate the virtuous cycle within the tourism industry.

### The coordinated development pathway of green urbanization and tourism competitiveness

#### The division of coordinated development types

**Figure 10:**
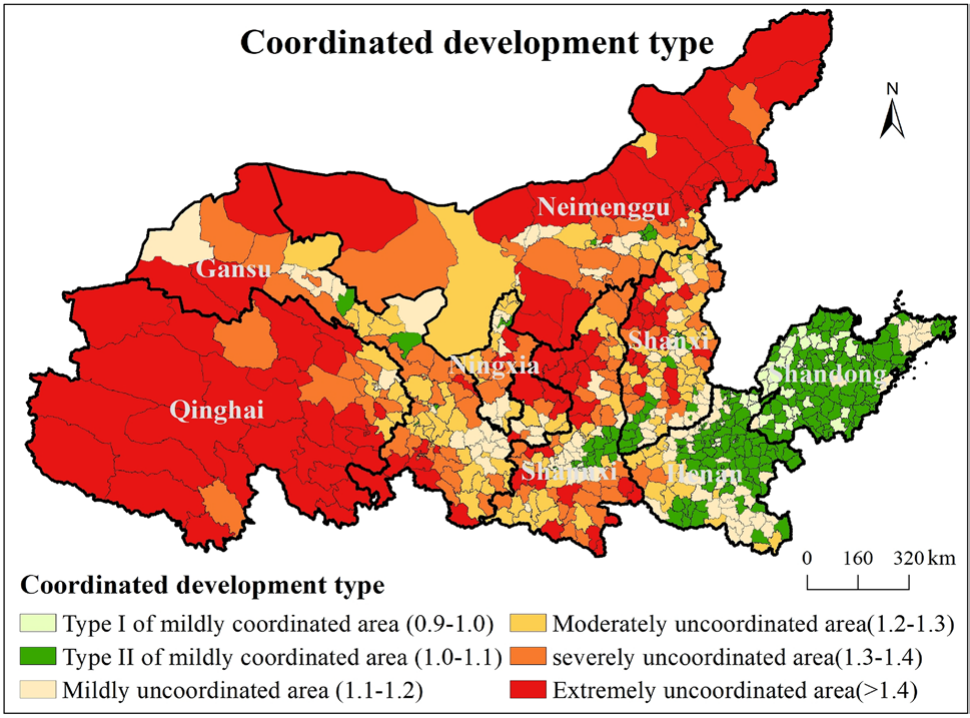
Results of classification of coordinated development types.

According to the spatial zoning method of coordinated development type, 735 counties and districts are divided into 6 types of districts. That is, mild coordination zone type I (52), mild coordination zone type II (231), mild discoordination zone (132), moderate discoordination zone (124), severe discoordination zone (124), and extremely discoordination zone (103) (Fig. 10). Take a closer look:

(1) Type I of mild coordination area and type II of mild coordination area. Among them, type I of mild coordination area represents the tourism competitiveness value of counties slightly less than the green urbanization. This type can be subdivided into type I of double-lag coordination area (both tourism competitiveness and green urbanization level are small) and type I of double-high coordination area (both tourism competitiveness and new urbanization level are large). The tourism competitiveness value of type II of mild coordination area is slightly greater than that of green urbanization. This type area can also be subdivided into type II of double-lag coordination and type II of double-high coordination. Type I of double-lag coordination area and double-lag coordination type II are mainly distributed in plain counties in the middle of the YRB, mountain counties and agricultural counties in the east region. Type I of double-high coordination area and type II of double-high coordination area are principally distributed in the economically developed region of the Guanzhong Basin and Fenhe River Basin, the central and western Henan Province (Luoyang, Zhengzhou, Pingdingshan, and Xuchang), the southern coast of Shandong Province (Rizhao, Qingdao, and Weihai), and Jinan metropolitan area.

(2) Mildly incongruous area. In this type of area, the ratio between the standardized value of tourism competitiveness and the standardized value of green urbanization level is 1.1–1.2, and the overall situation is slightly uncoordinated (tourism competitiveness is strong, but green urbanization is relatively weak). This type of area is principally distributed in the surrounding regions of the provincial capitals Xining and Yinchuan City, the tourist cities in northern Gansu Province, the mountainous counties of Liupanshan Mountain in Gansu Province, the counties around Xi 'an Metropolitan area, the Qinling Mountains, the Zhongtiao Mountains and Taihang Mountains in Shanxi Province, the mountainous counties in the northwest of Henan Province, the plain counties in southern Henan Province, and the coastal counties in the southeast of Shandong Province. According to its characteristics, it can be divided into three categories: (1) economically developed cities around provincial capitals; (2) Mountainous and coastal tourist cities with relatively weak economic development level but rich natural tourism resources; (3) Western tourist cities with relatively weak economic development level but rich natural resources and cultural heritage.

(3) Moderate dissonance area. This type of area is in a moderately uncoordinated state (standardized value of tourism competitiveness/standardized value of green urbanization level = 1.2–1.3). Compared with the mildly uncoordinated area, the gap between tourism competitiveness and the green urbanization level is larger, and the development level of tourism competitiveness is better than that of green urbanization. This type of area is principally distributed in the central region of the YRB, including the areas around the provincial capitals Xining City, Lanzhou City and Yinchuan City, the tourist cities in central and northern Gansu Province, Alashan Left Banner in central Inner Mongolia, Yulin City in the east of the Mu Us Desert, the Qinling Mountains, the mountains in central and eastern Shanxi Province, the counties in the western Henan Mountains and the counties in the southern Henan mountains.

(4) Severe dissonance area and extreme dissonance area. The severely uncoordinated area represents that the standardized value of tourism competitiveness is much higher than that of green urbanization (the ratio between the two is 1.3–1.4). The extremely uncoordinated areas represent that the standardized value of tourism competitiveness is much higher than that of green urbanization (the ratio between the two is greater than 1.4). The two types of regions coincide with each other in spatial distribution and are clustered, mainly distributed in large scale in most of Qinghai Province, the northern border area of Inner Mongolia, the eastern part of the Mu Us Desert in northern Shaanxi Province, the western mountainous area of Shanxi Province, as well as the economically underdeveloped inter-provincial border areas and remote mountainous areas with inconvenient transportation.

#### Strategies for coordinated development

(1) Mild coordination zone Type I and mild coordination zone type II. Type I of double lag coordination and type II of double lag coordination should make use of economic advantages and characteristic advantageous industries to promote the optimization, transformation and upgrading of industrial structure and drive the local economy. Improve people's living standards, improve the eco-environment, and make every effort to promote the construction of green urbanization. At the same time, tourism resources with local characteristics should be fully tapped to develop highly attractive tourism projects. The government should guide financial funds and social funds to invest in the development of tourism and the construction of related supporting industries, and promote the construction of tourism infrastructure and related supporting industries of public service facilities. Type I of double-high coordination and type II of double-high coordination should rely on rich city sightseeing resources and natural tourism resources, encourage cultural and tourism integration and innovation and increase tourism investment, improve the training system of tourism practitioners, strengthen the construction of tourism colleges and universities, improve the construction of tourism transportation, tourism catering, continue to innovate and develop, do a good job in "tourism+", Actively promote the transformation and upgrading of the tourism industry, and promote the further development of new urbanization with tourism innovation. In terms of the development strategy of new urbanization, we should improve the medium and long-term planning of new urbanization, and realize coordinated and intensive development among resources, industries, cities, management and regional development according to the comprehensive supply level of regional water resources, land resources, energy and mineral resources. Abolish the household registration system that separates urban and rural areas, establish the withdrawal and compensation mechanism of the right to use rural housing land, and establish and improve the urban and rural public service and social security system. We will improve cross-regional mechanisms for compensating and balancing the occupation and subsidy of resource protection, and promote coordinated development between urban development, resource exploitation and utilization, farmland protection and ecological protection. We will continue to build a new type of smart city that puts people first and develops harmoniously in the fields of science, education, culture, health and sports, so as to provide support and guarantee for improving the comprehensive competitiveness of tourism.

(2) Mildly incongruous zone. This type of area indicates that tourism competitiveness is strong, but green urbanization is relatively weak. In terms of green urbanization development strategy, emphasis should be placed on promoting green urbanization construction, developing moderate, intensive, green, and sharing type of towns. With ecology as the carrier, ecological and environmental protection mechanism should be established, and systematized, standardized and market-oriented ecological compensation mechanism should be gradually established. Formulate policies for the development of circular economy, and gradually establish a mechanism for the full coverage of resource recycling. We will formulate policies to support ecological industries, give priority to the development of resource-saving and environment-friendly industries, and encourage the development of high-tech and service industries with low dependence on resources and high added value. In terms of tourism competitiveness development strategy, this type of area is generally rich in natural tourism resources (mountain scenery, forest landscape, coastal landscape), urban sightseeing scenery and cultural relics, and the overall development level of tourism is also high. In the future, we should continue to improve the training system of tourism practitioners, strengthen the construction of tourism colleges and universities, and improve the construction of tourism transportation and tourism catering. Continue to innovate and develop, do a good job in "tourism+", actively promote the transformation and upgrading of the tourism industry, and promote the development of green urbanization with tourism innovation.

(3) Moderately uncoordinated zone. Compared with the mildly uncoordinated area, the gap between the tourism competitiveness and the green urbanization is larger, and the tourism competitiveness level is better than the green urbanization. In terms of tourism competitiveness development strategy, the tourism competitiveness of this type of area is strong. It should continue to improve the training system of tourism practitioners, strengthen the construction of tourism colleges, improve the construction of tourism transportation, tourism catering, and actively promote the transformation and upgrading of the tourism industry. In terms of the green urbanization development strategy, we should pay more attention to promoting the construction of new urbanization, pay attention to grasp their own advantages and characteristics, build a system of urban linkage and urban–rural complementarity, focus on creating characteristic towns with characteristic industries as the core, in order to promote the construction of new urbanization, in the process of promoting new urbanization, explore their own advantages, and gradually promote the development of industry. And then promote the improvement of tourism innovation ability. Mountain counties should develop the towns of moderate type, intensive type, green type, and sharing type, rationally planning and regulating the speed and scale of city extension, and build a protective and cooperative land use pattern through the planning and control of main functional zones. The speed of land urbanization should match the needs of population and industrial development, and the degree of intensive use and cohesion of urban land should be improved. We will strictly regulate market access policies for high-polluting and energy-intensive enterprises, and guide and foster low-carbon and green industries. In addition, with the ecology as the carrier, we will establish a mechanism for protecting the ecological environment, and gradually establish a systematic, standardized and market-based mechanism for compensating for ecological losses. Formulate a circular economy development policy, and gradually establish a full coverage of resources recycling mechanism. We will formulate policies to support ecological industries, give priority to the development of resource-saving and environment-friendly industries, and encourage the development of high-tech and service industries with low dependence on resources and high added value.

(4) Severely incongruous zone and extremely incongruous zone. The tourism competitiveness and green urbanization of these two types of areas are both in the primary stage of development, and the tourism competitiveness is better than that of green urbanization. In terms of tourism development, construction and competitiveness improvement, first of all, we should introduce successful tourism development experience of typical counties in central and eastern regions, formulate short and medium term development plans to promote tourism development, construction and competitiveness improvement according to regional advantages and characteristics, and guide the gradual and orderly development of regional tourism. Fully tap the tourism resources with regional characteristics, develop highly attractive tourism projects, and expand the influence of the tourism industry. We should improve the training system for tourism professionals, strengthen the construction of tourism colleges and universities, strengthen the construction of tourism transportation and tourism catering, continue to innovate and explore, and actively promote the development of tourism industry and its related supporting industries. In terms of green urbanization development strategy, first, establish a market-oriented mechanism for ecological industry cultivate based on the market. There are enormous discrepancy in resource endowment and industrial base among economically underdeveloped counties. According to the "goose array theory" in economics, each economy should rely on its own resource advantages to promote the upgrading and change of industrial structure, and form a "high-end, high-quality, high-tech, low-carbon and ecological green industry system, so that green industry can become a new engine. Second, with the support of science and technology, the establishment of ecological energy technology innovation mechanism. Scientific and technological progress and independent innovation should be taken as an important support and way to accelerate the transformation of economic development mode, focusing on the commonalities of pillar industries, strategic emerging industries and key industrial clusters and key technological breakthroughs. Third, we need to establish a mechanism for protecting the eco-environment, with ecology as the carrier. Gradually establish a systematic, standardized and market-oriented ecological compensation mechanism. Formulate development policies for the circular economy, and gradually establish a comprehensive mechanism for the recycling of resources. We will formulate policies to support ecological industries, give priority to the development of resource-saving and environment-friendly industries, and incite the cultivate of high-tech industries with low dependence on resources and high added value.

## Conclusions

Exploring the spatial cooperative relationship and interaction mechanism between green urbanization and tourism competitiveness is of great significance for promoting urban sustainable development. In this study, the green urbanization level and tourism competitiveness in the YRB were scientifically evaluated based on multi-source data, and then the interactive mechanism and collaborative development path of green urbanization and tourism competitiveness were deeply discussed. The main conclusions are as follows:

(1) The green urbanization shows a multi-core circle structure with provincial capitals, prefecture-level city districts and economically developed counties as the high-value centers, and the trend of agglomeration distribution is significant. The tourism competitiveness presents a polycentric spatial structure. Additionally, a significant spatial coupling relationship between green urbanization and tourism competitiveness exists in the YRB.

(2) Factors such as green urbanization level, social progress, population development, infrastructure construction, economic development quality, and eco-environmental protection all have significant effects on the tourism competitiveness. Tourism competitiveness, service competitiveness, location competitiveness, resource competitiveness, market competitiveness, environmental influence and talent competitiveness have a significant impact on the green urbanization level.

(3) The green urbanization has multiple supporting effects on tourism competitiveness. The development of population development provides population support for industrial development, tourism and related service industries. Social progress and infrastructure construction play a basic supporting role in tourism competitiveness. Economic development quality provides strong financial support for social development and infrastructure construction and indirectly promotes the tourism competitiveness. Eco-environmental protection can promote the quality of the eco-environment and improve tourists' travel experience and impression. Tourism competitiveness has a strong driving effect on green urbanization. The improvement of tourism competitiveness can provide a continuous driving force for economic development quality, industrial transformation, population development, social progress, infrastructure construction, eco-environmental protection, and thus promote the steady development of green urbanization. Tourism competitiveness and green urbanization complement each other. Tourism competitiveness can promote the development of green urbanization, and the improvement of green urbanization level can support tourism competitiveness. The two complement each other, forming a virtuous circle of tourism competitiveness and green urbanization, and ultimately promoting the high-quality development and sustainable development of counties together.

(4) According to the spatial division method of coordinated development types, 735 counties and districts were divided into 6 t0ypes, namely, type I of mildly coordinated area, type II of mildly coordinated area, mildly uncoordinated area, moderately uncoordinated area, severely uncoordinated area and extremely uncoordinated area. Then, according to the development situation of different types of areas, it puts forward the coordinated development strategy of tourism competitiveness and green urbanization of each type areas.

This study systematically explores the spatial coupling relationship and interaction mechanism between green urbanization and tourism competitiveness, which is of great significance for promoting sustainable urban development. However, there are still some limitations and disadvantages. First, due to the lack of indicators related to infrastructure competitiveness before 2022 (vector data of high-speed rail sites, airport sites and highway networks) and service competitiveness before 2016 (number of star-rated hotels, travel agencies and public toilets in the counties). Therefore, only cross-sectional data (2022) was used in this study, but panel data was not used. Second, due to the difficulty in obtaining county index data, the evaluation index system of county green urbanization and tourism competitiveness proposed in this study needs to be further improved. In the future, remote sensing technology, big data technology and survey questionnaire can be used to obtain more evaluation index data, so as to improve the evaluation index system of county green urbanization and tourism competitiveness. Third, this study has not considered the negative impact factors on green urbanization and tourism competitiveness, as well as the negative impact factors of the coupling relationship between the two, which is what future research needs to pay attention to. Fourth, although this study attempts to systematically analyze the theoretical interaction mechanism between green urbanization and tourism competitiveness, its theoretical interaction mechanism has not been fully revealed. Therefore, the theoretical interaction mechanism between green urbanization and tourism competitiveness remains the focus and difficulty of future research.

## Data Availability

The data presented in this study are available on request from the corresponding author. The data are not publicly available due to privacy restrictions.
